# Supplementary material for: How local reference panels improve imputation in French populations
Source: Sci Rep. 2024 Jan 3;14:370. doi: 10.1038/s41598-023-49931-3 (PMC10764714; doi:10.1038/s41598-023-49931-3)
Supplement: Supplementary file 1 — Supplementary Information. [file 41598_2023_49931_MOESM1_ESM.docx]

Supplementary Materials

How local reference panels improve imputation in French populations

Anthony F. Herzig^1,*^, Lourdes Velo‐Suárez^1,2^, FrEx Consortium^1^, FranceGenRef Consortium^3^, Christian Dina^4^**,** Richard Redon^4^, Jean-François Deleuze^5,6^, Emmanuelle Génin^1,2^

**1 :**Univ Brest, Inserm, EFS, UMR 1078, GGB, Brest, France

**2 :** CHRU Brest, Brest, France

**3 :** LABEX GENMED, Centre National de Recherche en Génomique Humaine, Evry, France

**4 :**Nantes Université, CHU Nantes, CNRS, INSERM, l’institut du thorax, Nantes, France

**5 :**Université Paris-Saclay, CEA, Centre National de Recherche en Génomique Humaine (CNRGH), Evry, France

**6**: Fondation Jean Dausset - Centre d’Etude du Polymorphisme Humain (CEPH), Paris, France

* Corresponding Author - anthony.herzig@inserm.fr

***Details on the utilisation of imputation servers:***

We carried out a set of tests of the performance of two imputation servers (Michigan and Sanger). We were able to vary the choice of variants to represent different possible genotyping arrays (Supplementary Figure 1). We tested the following arrays: The Illumina Core Exome array (ICE), the Axiom Precision Research Array (PRMA), and the UK Biobank imputation array (UKBB). Furthermore, we could also test different imputation software (MINIMAC4^1^ and PBWT^2^). For both servers, EAGLE2^3^ was used as phasing software as this is currently the only choice available on the Michigan server. These results are presented in Figure 1. We found that the genotyping array that clearly gave the most accurate imputed genotypes was UK Biobank Axiom array. Imputation accuracy was overall slightly higher on the Michigan server compared to the Sanger server, probably due to the difference in imputation algorithm being used (MINIMAC4 or PBWT, respectively).

***Details on Quality Control using VCFProcessor***^4^ ***using the QC1078 setting:***

Genotypes were set to missing when:

- Depth (DP) < 10
- Genotype Quality (GQ) < 20

Subsequently, variants were excluded (for all individuals) using the following criteria for various summary statistics (measured across all samples) generated by GATK v.3.8^5^.

- Allele Balance for Heterozygous calls (ABHet) outside of the range [0.25,0.75]
- Quality-By-Depth < 2
- MQRankSum < - 12.5 (Z-score from Wilcoxon rank sum test of Alt vs. Ref read mapping qualities)
- Mapping Quality (MQ) < 40 for SNPs or < 10 for INDELS.
- Strand Bias odds ratio > 3 for SNPs or > 10 for INDELS
- Fisher’s exact test for strand bias (phred scaled p-value) > 60 for SNPs or > 200 for INDELS
- HQRatio < 0.8
- Inbreeding Coefficient (estimated) < -0.8
- Callrate < 0.9


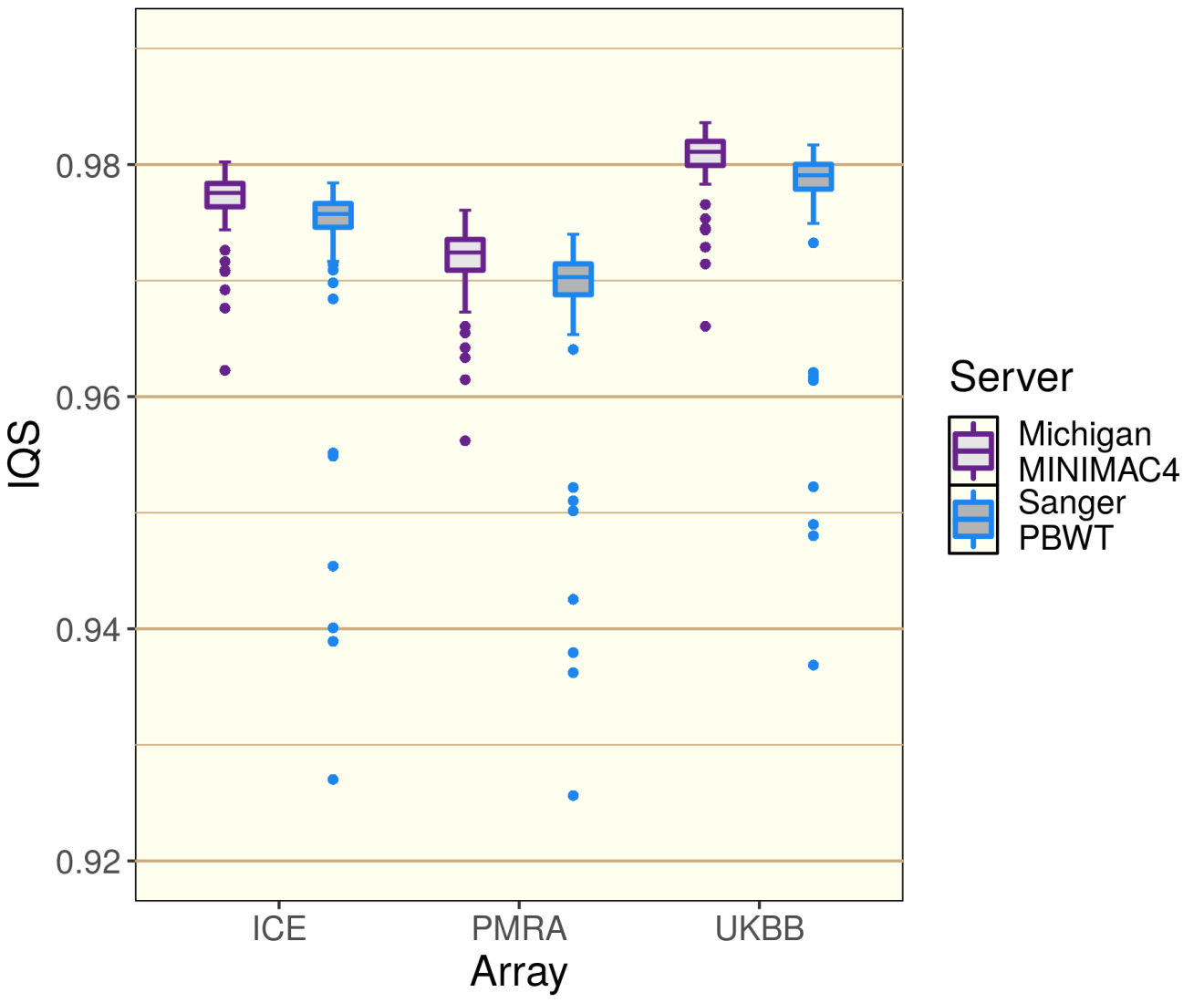


*Supplementary Figure 1: Imputation accuracy of different servers and arrays. For each individual, array positions were extracted, sent to an imputation server before calculating an individual Imputation Quality Score (IQS) by comparing the sequenced non-array positions against imputed counterparts. ICE: Illumina Core Exome array, PRMA: Axiom Precision Research Array, UKBB: UK Biobank imputation array.*


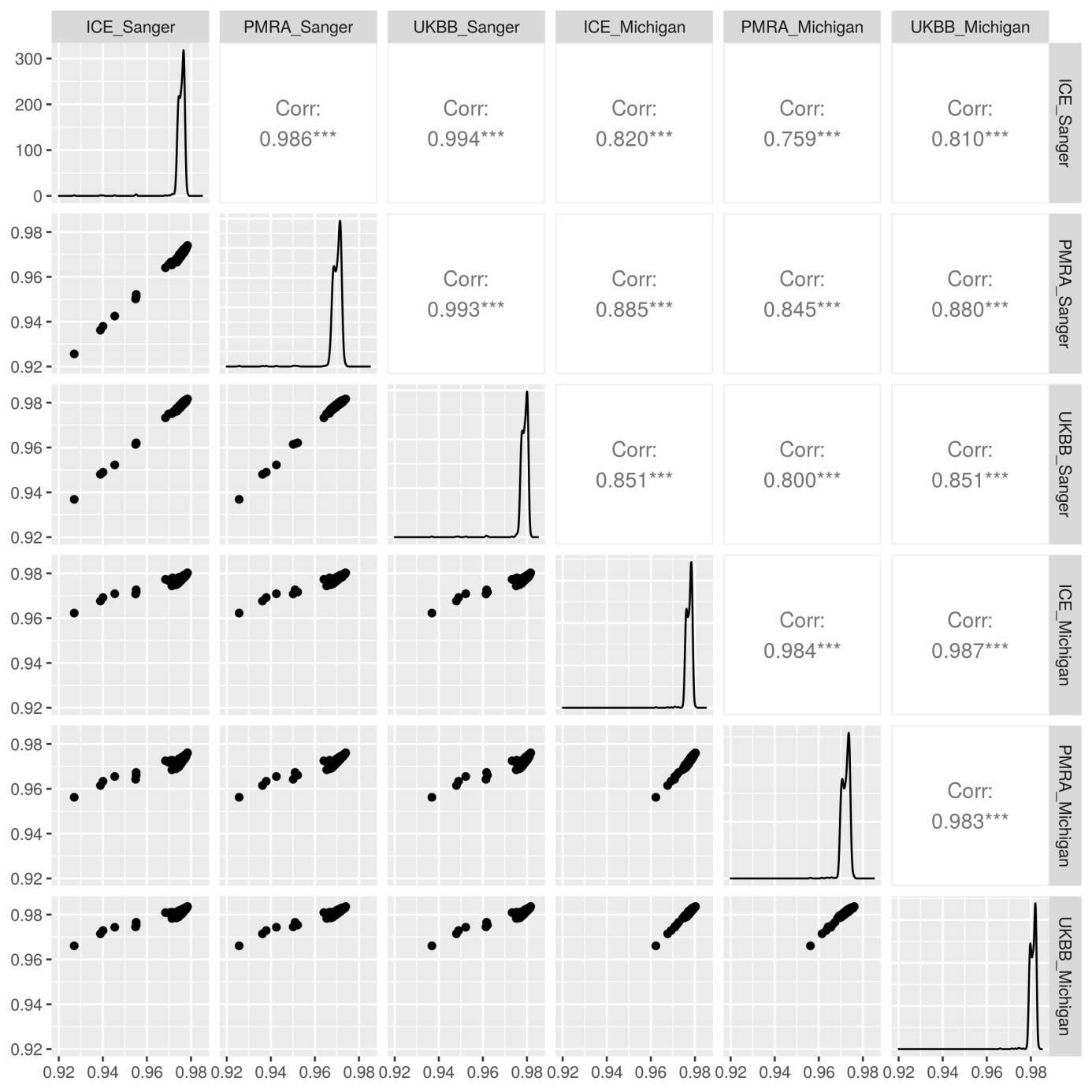


*Supplementary Figure 2: The same individuals are imputed well or poorly, irrespective of the imputation server or array chosen. Corr = Correlation. ICE: Illumina Core Exome array, PRMA: Axiom Precision Research Array, UKBB: UK Biobank imputation array.*


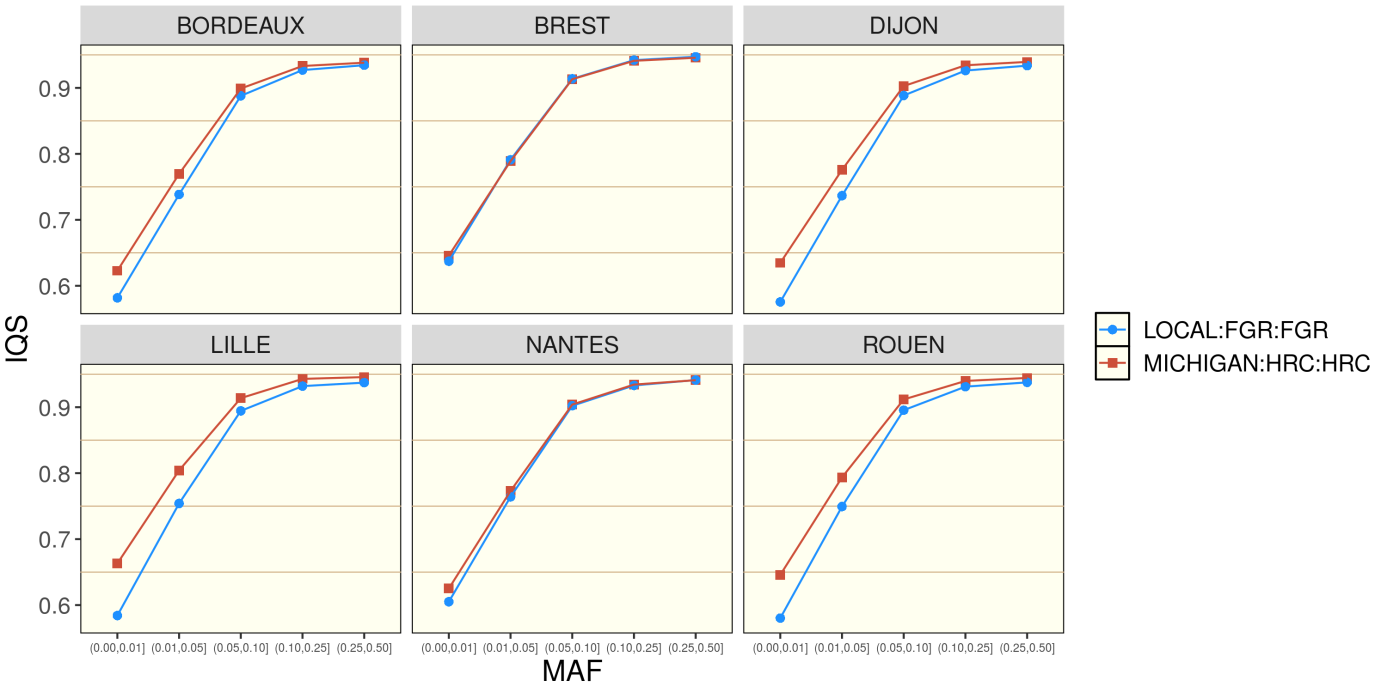


*Supplementary Figure 3 - MICHIGAN:HRC:HRC vs LOCAL:FGR:FGR. Mean per-variant IQS scores for five different minor allele frequencies (MAF) bins are presented. MAF was measured separately in each city by counting the observed number of minor alleles in each group of FrEx.*

**
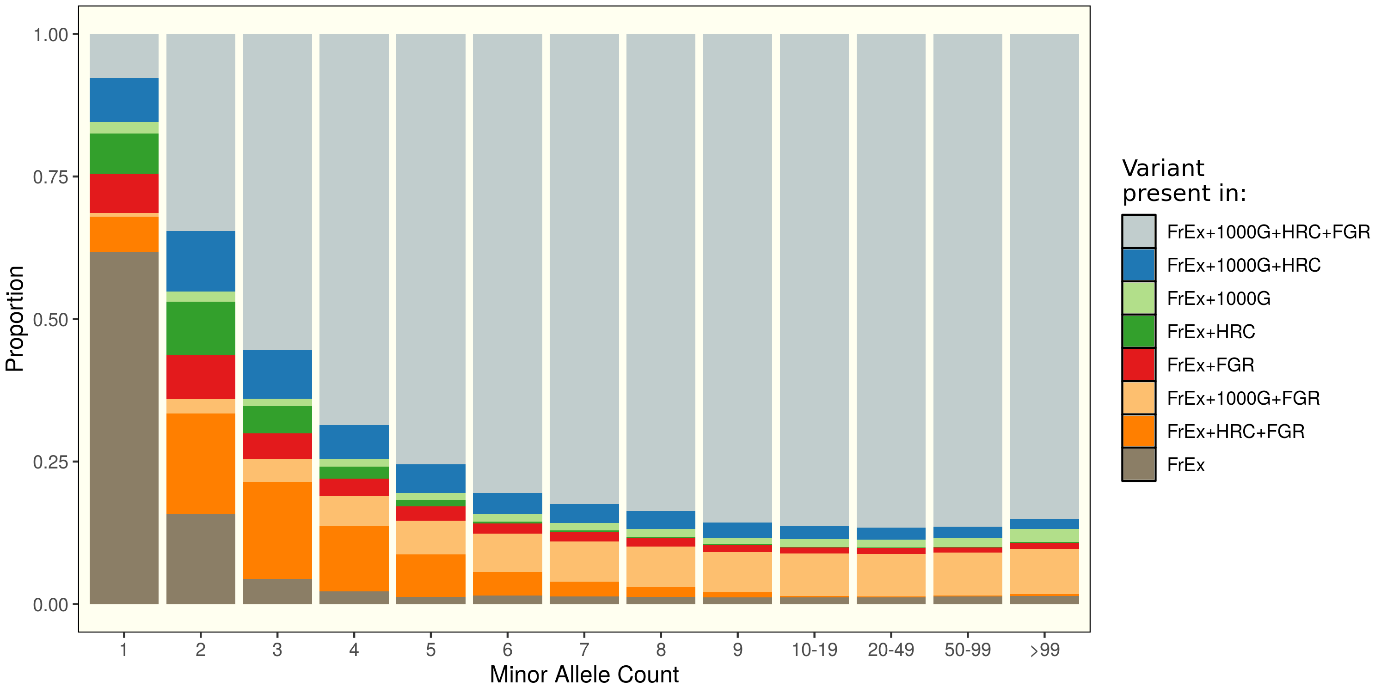
**

*Supplementary Figure 4. Proportions of variants which are observed in FrEx and other datasets split by different Minor Allele Count (MAC) bins measured in FrEx. For example, the group ‘FrEx’ represents the variants that are observed in FrEx but nowhere else, ‘FrEx+1000G+HRC+FGR’ represents the variants observed in all 4 datasets, and ‘FrEx+FGR’ represents the variants observed only in the French datasets FrEx and FGR.*

***
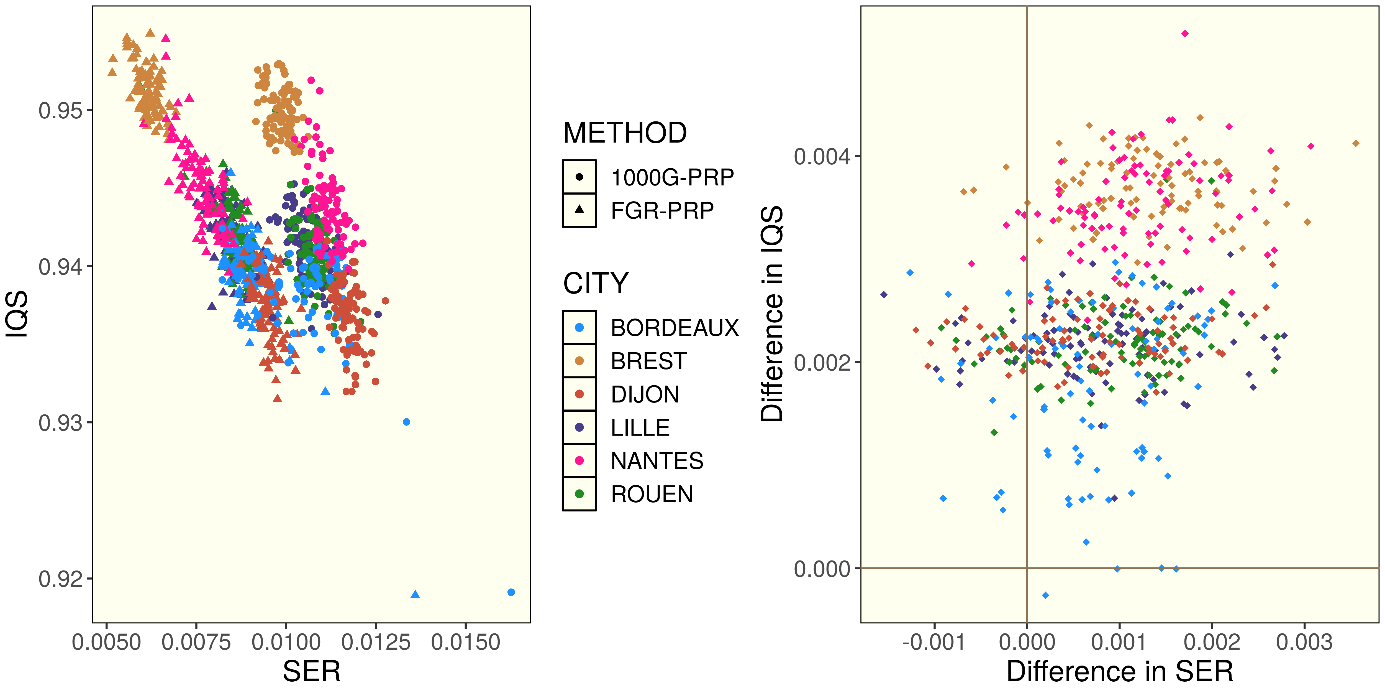
***

*Supplementary Figure 5. Comparison of imputation and phasing accuracy results using either FGR (FGR-PRP) or 1000G (1000G-PRP) as a phasing reference panel for FrEx. Left: Individuals approximated Switch-Error Rate (SER) statistics against individual Imputation Quality Score (IQS) statistics. Clear correlation was observed in both clouds of points (FGR-PRP or 1000G-PRP). Right: to show that the change of PRP has a more pronounced consequence on the individuals coming from Brest and Nantes, the differences in the SER and IQS statistics from the Right panel are plotted. The individuals from Brest and Nantes have noticeably greater differences (representing greater improvement) in both statistics when moving from 1000G-PRP to FGR-PRP.*


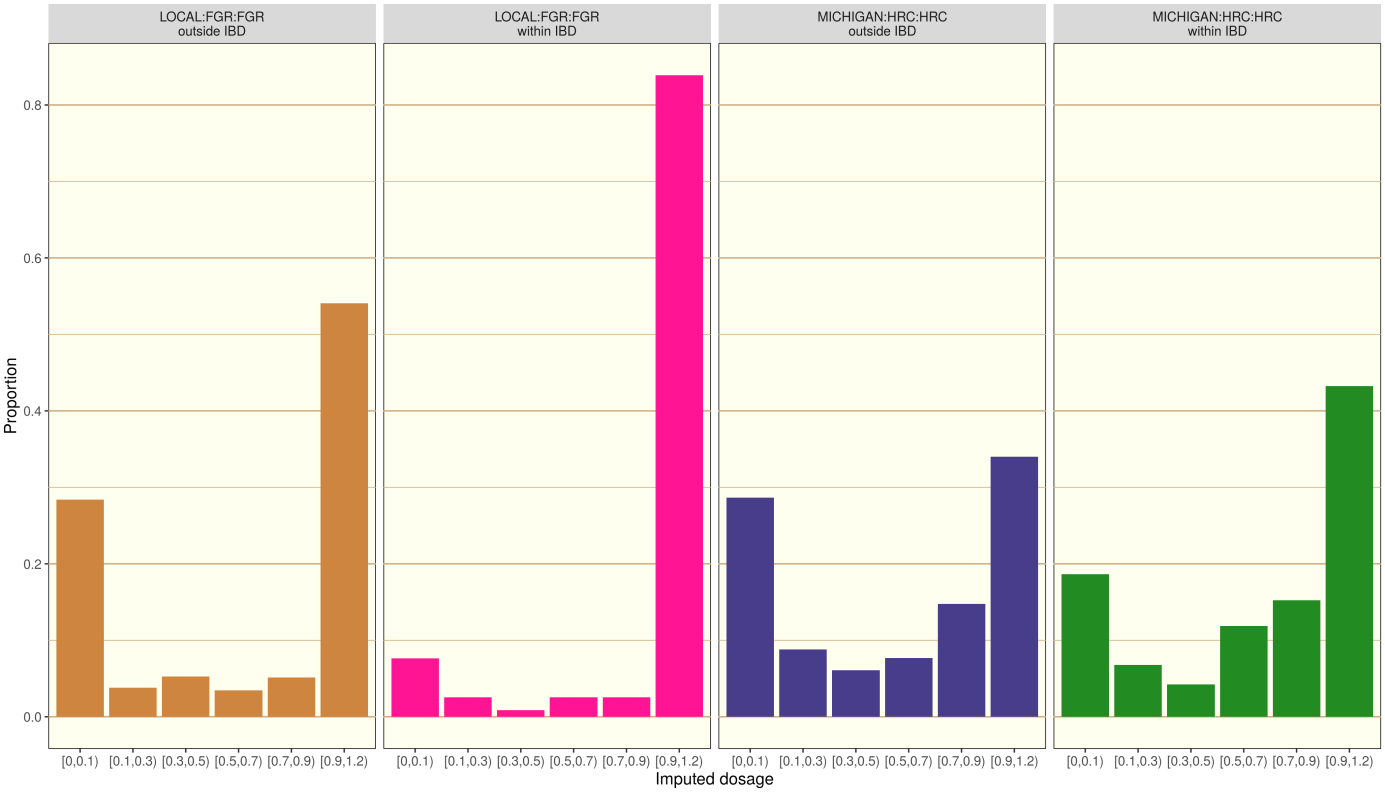


*Supplementary Figure 6 - Imputed dosage of heterozygote genotypes of rare-variants (MAF<0.01) obtained for FrEx individuals from Brest for two different sets of variants, those inside and outside of the long IBD segments shared with FGR clusters 1 and 2 and for two different imputation strategies (LOCAL:FGR:FGR and MICHIGAN:HRC:HRC). Imputed dosage is the expected minor allele count for each imputed genotype based on the posterior imputation probabilities for the genotypes with zero minor alleles (AA), 1 minor allele (Aa) and 2 minor alleles (aa). For the set of heterozygote genotypes investigated here, the correct imputed dosage should be equal to 1. Imputed dosages such as 0.5 that are distant from the set {0,1,2} reflect a high level of uncertainty, and hence inaccurate imputation. No dosage values were observed outside of the range [0-1.2].*

**
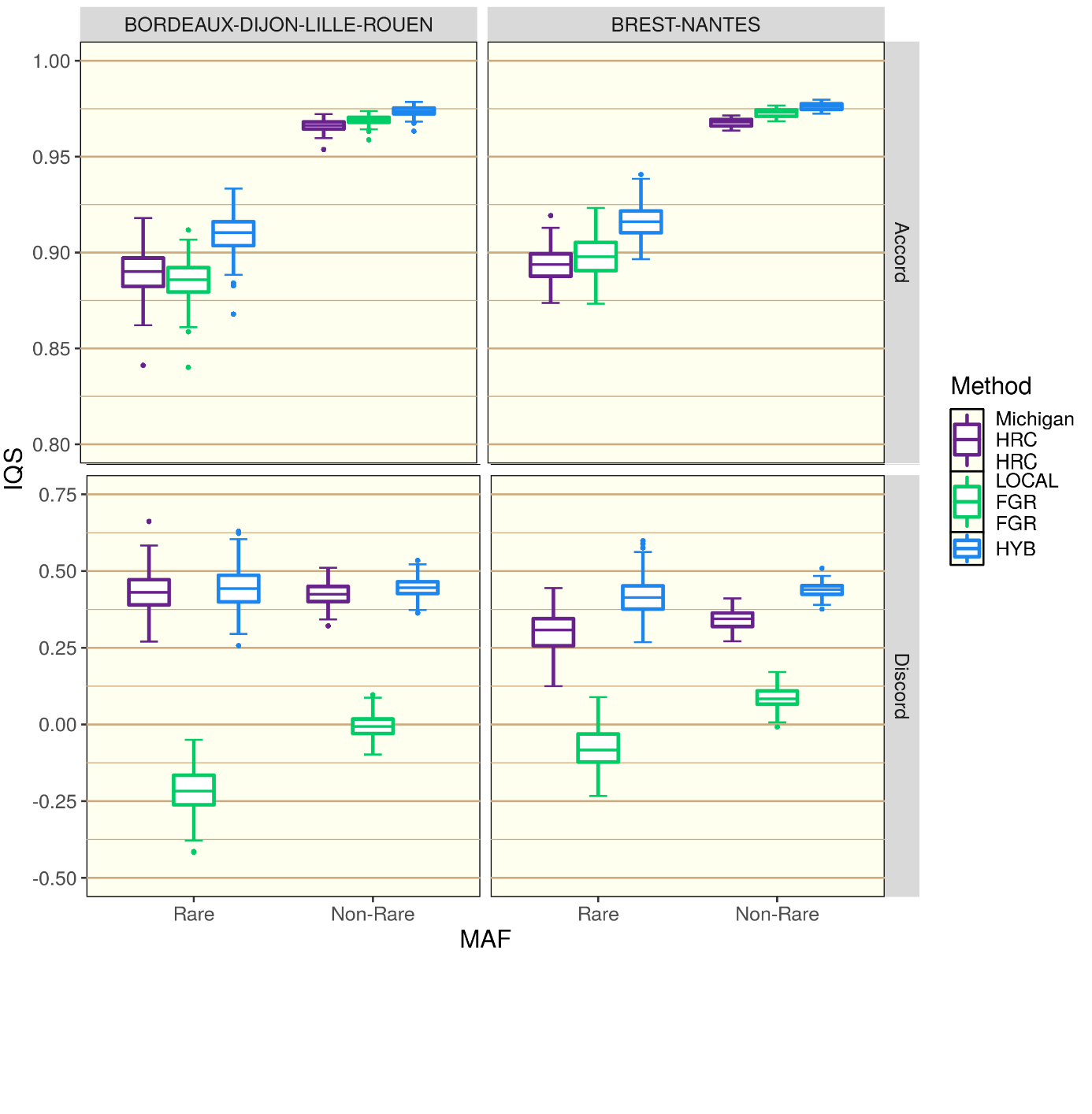
**

*Supplementary Figure 7. Individual IQS scores calculated for different variant sets. Firstly dichotomising between rare and non-rare variants (MAF below of above 0.01 measured in FrEx) and secondly dichotomising between whether imputation runs MICHIGAN:HRC:HRC and LOCAL:FGR:FGR were in agreement or not (Accord or Discord, respectively) regarding the most likely genotype. Note the difference y-axis ranges on top two and bottom two panels.*

*
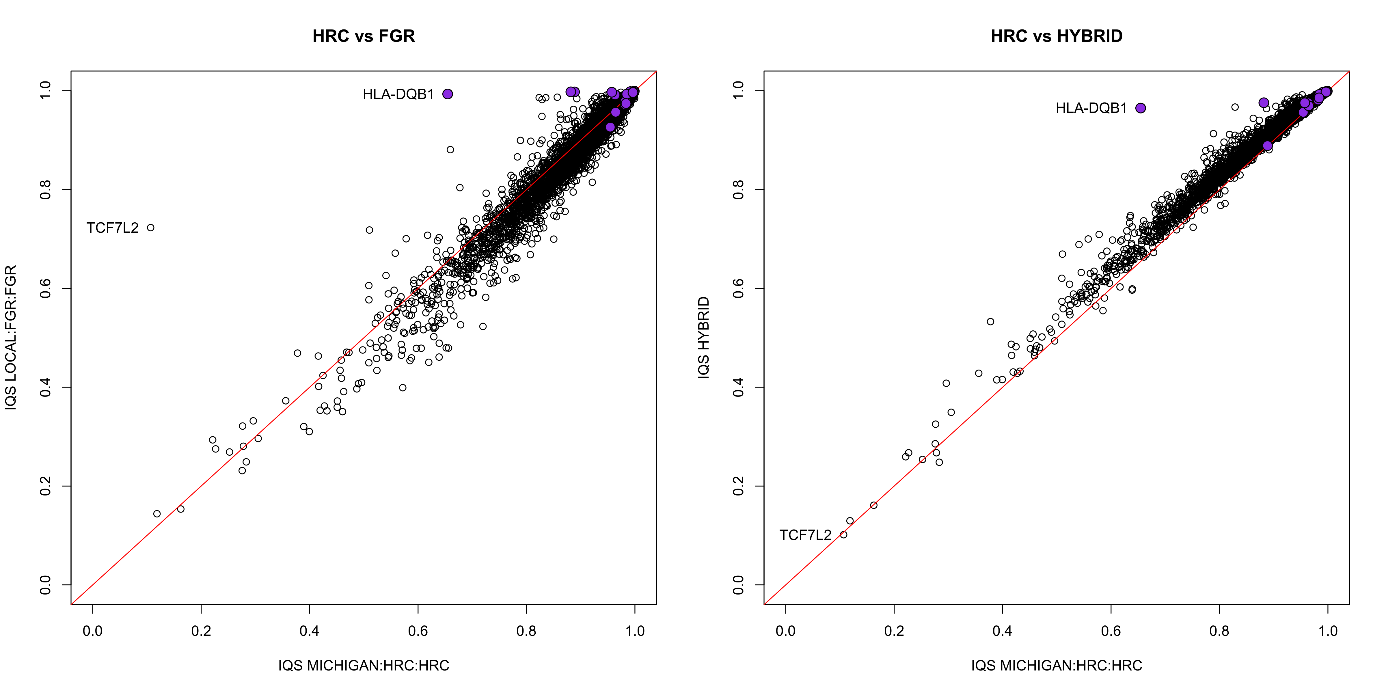
*

*Supplementary Figure 8a: Imputation Quality Scores (IQS) calculated per-gene across all individuals of FrEx and compared between different imputation strategies. Only genes with at least 5 polymorphic variants in the truth dataset were considered. Two genes observed to differentiate substantially from the red equality line in the left plot are highlighted. HLA-genes are highlighted with purple points.*

*
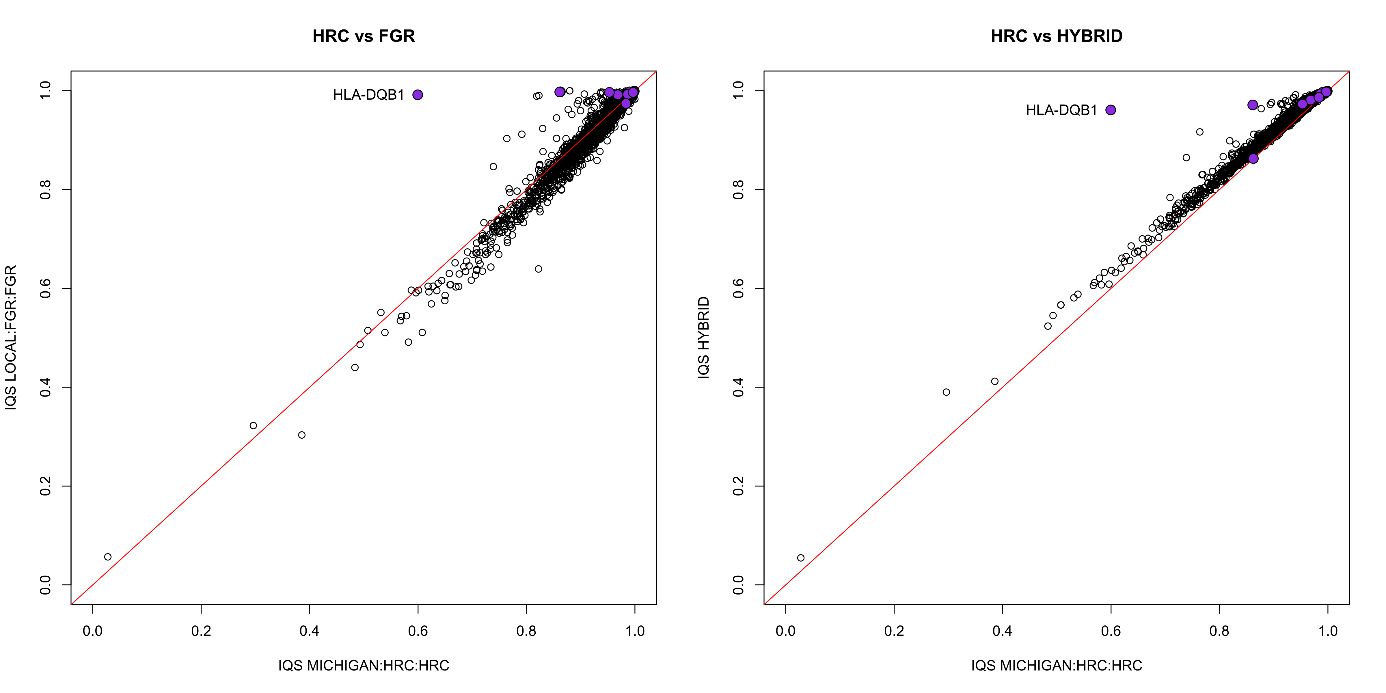
*

*Supplementary Figure 8b: As 8a but only including variants with a minor-allele frequency above 0.05.*

*
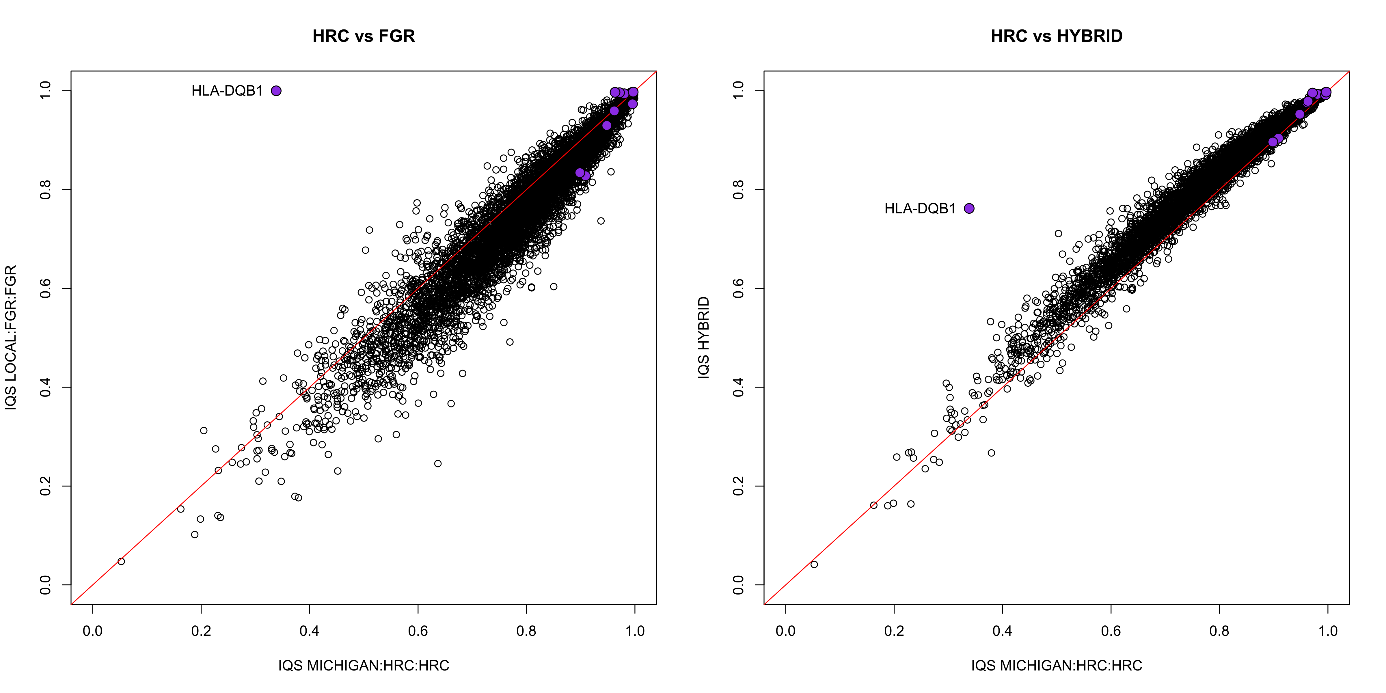
*

*Supplementary Figure 8c: As 8a but only including variants with a minor-allele frequency below 0.05.*

| **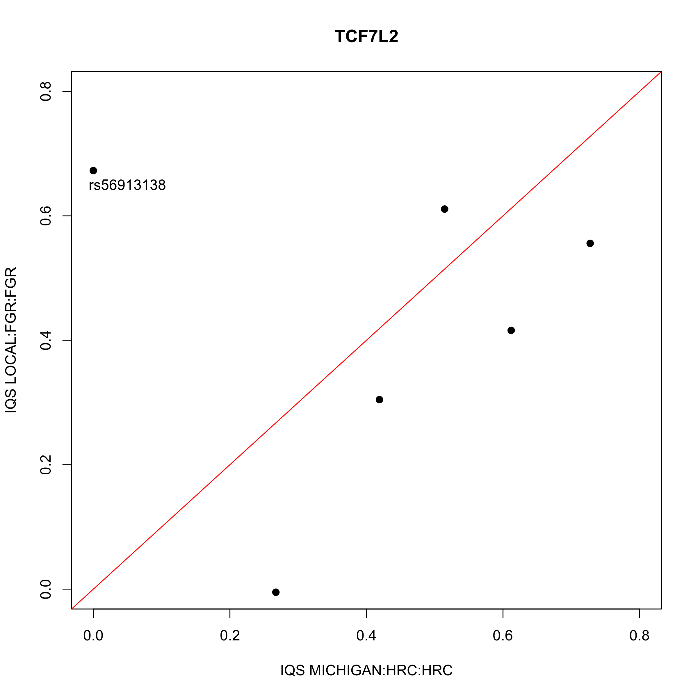** | **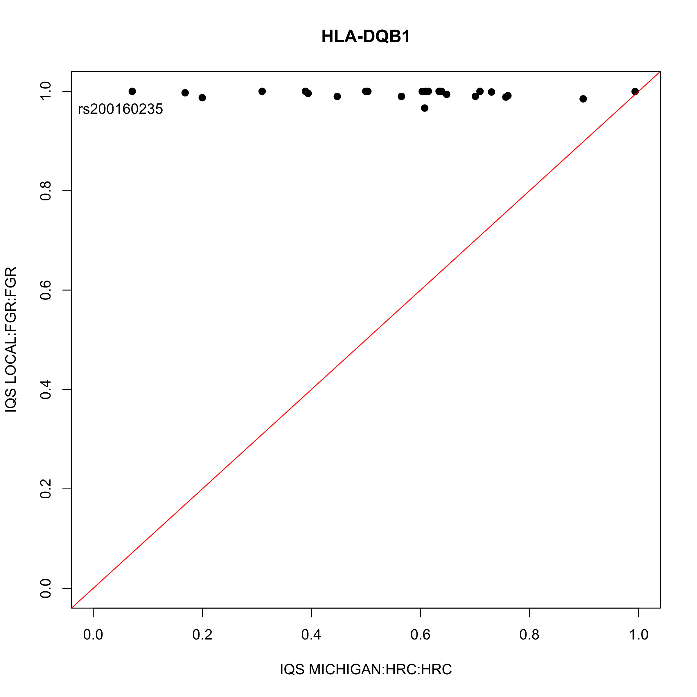** |
| --- | --- |

*Supplementary Figure 9a-b: Per-variant Imputation Quality Scores (IQS) for genes TCF7L2 and HLA-DQB1 for the imputation strategies LOCAL:FGR:FGR and MICHIGAN:HRC:HRC. Two variants are highlighted and their IQS tables are given in detail in Supplementary Tables 1a-b.*

| *Truth (rs56913138)*  *Imputed*  *MICHIGAN:HRC:HRC*  *LOCAL:FGR:FGR* | *AA* | *Aa* | *aa* |
| --- | --- | --- | --- |
| $\Sigma$*P(G=AA)* | *320.932*  *270.580* | *194.976*  *26.185* | *29.997*  *0.100* |
| $\Sigma$*P(G=Aa)* | *0.068*  *49.491* | *0.024*  *156.481* | *0.003*  *6.985* |
| $\Sigma$*P(G=aa)* | *0*  *0.924* | *0*  *12.319* | *0*  *22.914* |

*Supplementary Table 1a: Table showing the correspondence between true genotypes for rs56913138 in the FrEx against the sum of posterior genotype probabilities (dosages) for the two imputation strategies MICHIGAN:HRC:HRC (blue) and LOCAL:FGR:FGR (red).*

| *Truth (rs200160235)*  *Imputed*  *MICHIGAN:HRC:HRC*  *LOCAL:FGR:FGR* | *AA* | *Aa* | *aa* |
| --- | --- | --- | --- |
| $\Sigma$*P(G=AA)* | *488.822*  *546* | *1.282*  *0* | *0*  *0* |
| $\Sigma$*P(G=Aa)* | *55.573*  *0* | *2.647*  *4* | *0*  *0* |
| $\Sigma$*P(G=aa)* | *1.579*  *0* | *0.072*  *0* | *0*  *0* |

*Supplementary Table 1b: As supplementary table 1a but for rs200160235.*

In these two tables, the raw data used to calculate IQS scores for two SNPs highlighted in Supplementary Figures 9a-b are given; to help explain why the two genes stand out in Supplementary Figures 8a-c. The difference in IQS for TCF7L2 is largely driven by just one variant, *rs56913138. In supplementary table 1a, f*or *rs56913138, we could see that the imputation is not particularly accurate with LOCAL:FGR:FGR but completely incorrect with MICHICAN:HRC:HRC, it is almost completely imputed as homozygous for the reference. This could well suggest a data quality issue in the HRC for the variant. Note that in the reference database gnomAD*^6^ *this variant has a minor allele-frequency of 0.202 in non-Finnish Europeans so it would seem unlikely that it would imputed with a frequency so low in FrEx.*

*In supplementary table 1b, for rs200160235 in HLA-DQB1, we see that the imputation was perfect with LOCAL:FGR:FGR, all four heterozygotes are correctly identified. But MICHIGAN:HRC:HRC did much less well. This variant has a minor allele-frequency of 0.0107 in gnomAD for non-Finnish Europeans. This is slightly higher than what we observe in FrEx (0.00364) but a lot less than what would be inferred from MICHIGAN:HRC:HRC (0.0556).*

*We can also note that the HYB strategy preserves the strengths of LOCAL:FGR:FGR for HLA-DQB1 but unfortunately for TCF7L2 HYB preferred the likely erroneous imputation of MICHIGAN:HRC:HRC; demonstrating the limitations of the strategy.*

|  | |  | |  |
| --- | --- | --- | --- | --- |
|  | **Eigen Decomposition** | |  | |
| **Eigen Vector 2** | **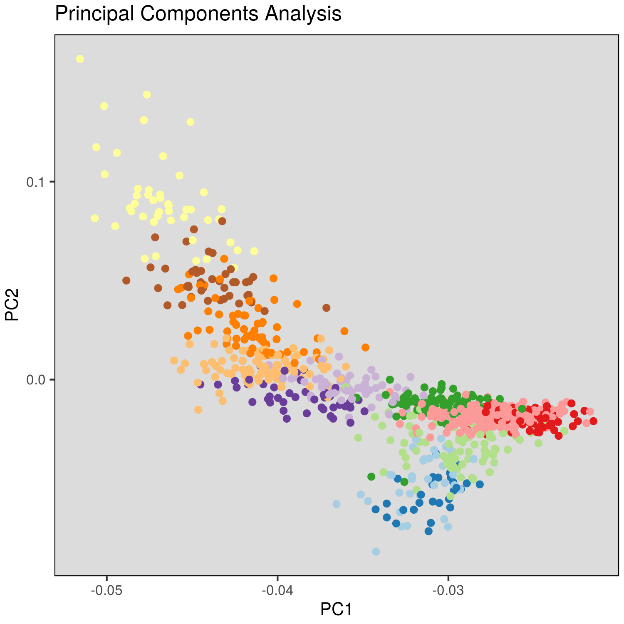** | | **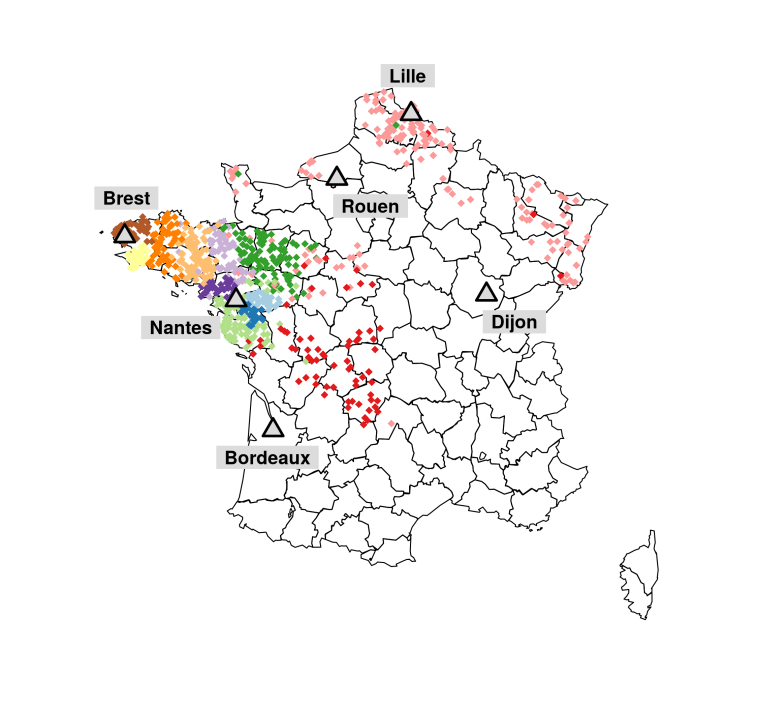** | |
|  | **Eigen Vector 1** | |  |  |

*Supplementary Figure 10. Eigen decomposition analysis of the IBD-sharing matrix in FGR (left). Colours represent the 12 clusters identified by finestructure*^7^ *and described in the main text. The correspondence with geographical locations is also given for comparison (right) where individuals from FranceGenRef are plotted as diamonds.*

**References**

1. Das, S. *et al.* Next-generation genotype imputation service and methods. *Nat Genet* **48**, 1284–1287 (2016).

2. Durbin, R. Efficient haplotype matching and storage using the positional Burrows-Wheeler transform (PBWT). *Bioinformatics* **30**, 1266–1272 (2014).

3. Loh, P.-R. *et al.* Reference-based phasing using the Haplotype Reference Consortium panel. *Nat Genet* **48**, 1443–1448 (2016).

4. Ludwig, T. E., Marenne, G. & Génin, E. VCFProcessor. http://lysine.univ-brest.fr/vcfprocessor/index.html. Accessed 08/10/2020. (2020).

5. DePristo, M. A. *et al.* A framework for variation discovery and genotyping using next-generation DNA sequencing data. *Nat. Genet.* **43**, 491–498 (2011).

6. Karczewski, K. J. *et al.* The mutational constraint spectrum quantified from variation in 141,456 humans. *Nature* **581**, 434–443 (2020).

7. Lawson, D. J., Hellenthal, G., Myers, S. & Falush, D. Inference of Population Structure using Dense Haplotype Data. *PLOS Genetics* **8**, e1002453 (2012).
